# Capacity of humic substances to complex with iron at different salinities in the Yangtze River estuary and East China Sea

**DOI:** 10.1038/s41598-017-01533-6

**Published:** 2017-05-03

**Authors:** Rujun Yang, Han Su, Shenglu Qu, Xuchen Wang

**Affiliations:** College of Chemistry and Chemical Engineering, Ocean University of China, Songling Road 238, Qingdao, 266100 P.R. China

## Abstract

The iron binding capacities (IBC) of fulvic acid (FA) and humic acid (HA) were determined in the salinity range from 5 to 40. The results indicated that IBC decreased while salinity increased. In addition, dissolved iron (dFe), FA and HA were also determined along the Yangtze River estuary’s increasing salinity gradient from 0.14 to 33. The loss rates of dFe, FA and HA in the Yangtze River estuary were up to 96%, 74%, and 67%, respectively. The decreases in dFe, FA and HA, as well as the change in IBC of humic substances (HS) along the salinity gradient in the Yangtze River estuary were all well described by a first-order exponential attenuation model: y(dFe/FA/HA, S) = a_0_ × exp(kS) + y_0_. These results indicate that flocculation of FA and HA along the salinity gradient resulted in removal of dFe. Furthermore, the exponential attenuation model described in this paper can be applied in the major estuaries of the world where most of the removal of dFe and HS occurs where freshwater and seawater mix.

## Introduction

Iron (Fe) is an essential micronutrient in aquatic systems. Both zooplankton and algae take up iron to carry out photosynthesis and formation of chlorophyll and carbohydrates^1, 2^. One study showed that iron was vitally importance for nitrogen fixation and phosphorus limitation in the north Pacific^3^. However, the concentration of Fe^3+^ in sea water is low because Fe^3+^ exists as Fe(OH)_3_, which is not soluble in sea water at pH 8.2^4^. More than 99.9% of dissolved iron exists in organic complexes^5–10^. Humic substances (HS), which account for most organic iron ligands^11, 12^, are the major organic matter component in natural waters, such as freshwater lakes and rivers, estuaries, and coastal waters (15–80%)^13–17^. Previous studies reported that HS could bind a large number of trace metals in the aquatic system, such as Al, Cu and Fe in seawater^18–21^. Land-derived HS acting as a natural iron chelators are important factors, affecting the availability of dFe for marine phytoplankton^11, 22–27^. Furthermore, HS reportedly acts as strong iron ligands in the ocean^12^, mostly in colloidal form, and holds a large quantity of trace metals in fresh water^20, 28, 29^. Therefore, HS tends to be flocculate when fresh water mixes with seawater in estuaries and salinity increases^12, 20, 29^. Earlier studies have demonstrated that the colloidal nature of HS would rapidly flocculate in electrolyte solutions and seawater^30–33^.

As a result, dissolved iron co-precipitates together with HS in estuaries^20, 29^. More than 90% of dissolved iron was deposited from a water mass when salinity increased from 0 to 30.4^29^. The decrease of HS may be responsible for scavenging of dFe in estuaries because the colloidal nature of HS-metal complexation flocculates with different ions as salinity increases in the estuary^20, 28^. Earlier studies have assumed that more than 99% of dFe and freshwater HS co-precipitated and were lost in estuaries^20^. However, approximately 20% of aquatic humic substances resist precipitation and remain dissolved in seawater^25, 26, 34, 35^. Therefore, the removal ratio of HS in an estuary would influence the ultimate input of iron to seawater.

The total dissolved iron bound by a unit mass of HS, which was first defined by Laglara *et al*. (2007) to be the iron binding capacity of HS (IBC), in different salinities was of great importance^11, 36^. Laglera *et al*.’s (2007) results indicated that the IBC of humic acid (HA) was 32 ± 2.2 nmol Fe/(mg HA), whereas the IBC for fulvic acid (FA) was 16.7 ± 2.0 nmol Fe/(mg FA)^11, 36^. They also indicated that the total dissolved HS decreased as the distance from the mouth of the Mercy River increased. Moreover, they reported that the structure of HS might change as salinity increases, so IBC may be influenced by salinity^37^. The structure of HA and the Fe-HS complexation could be different in solutions of different ionic strengths^18^. Therefore, the capacity of HS to complex with dFe could change along salinity gradients in estuaries. Furthermore, according to Krachler *et al*. (2015), terrestrial HS, which is mainly present in estuaries and coastal waters, decreases rapidly with increasing salinity as well as with distance from land^27^. Esteves *et al*. (2009) found that HS in coastal waters was mainly terrestrially sourced, whereas HS in open oceans was mainly marine-derived^38^. HS from terrestrial sources has more aromatic and less aliphatic structures than that from marine sources^38^. Fe-HS complexation principally involves carboxylates and phenolic groups in binding^39^. Terrestrial and marine HS may have different complexing capacities for Fe. Therefore, we propose that HS may have differences in its IBC depending on salinity gradients in estuarine systems. However, very little research has been carried out on the variation of IBC at different salinities.

This paper estimates the IBC of HS at different salinities in the Yangtze River and its estuary and develops a numerical model for dFe, HS and IBC at different salinities, so that the removal rate of HS and iron in estuaries, such as the Yangtze River estuary (YRE) and the East China Sea (ECS) can be calculated. The Yangtze River is the third largest river with a huge estuary, draining into the ECS, one of the largest marginal seas. The YRE is long and has a distinct salinity gradient, making it a good place to study the behavior of HS, Fe and the IBC where fresh water and seawater mix. The dFe, FA and HA concentrations along the salinity gradient, from the freshwater end-member of the YRE (salinity < 0.5) to the ECS (salinity > 34), were collected and determined. This work enabled us to optimize the parameters of the Cathodic Stripping Voltammetry (CSV) response for Fe-HS complexations. In these samples, we investigated the co-variation of dFe with HS in the low salinity area, and we developed numerical models of HS, dFe and the IBC in the YRE, which fit results from other studies of other estuaries well. To our knowledge, this study is the first attempt to model the removal of dFe, FA and HA along salinity gradients in the YRE or other estuaries.

## Methods

**Figure 1:**
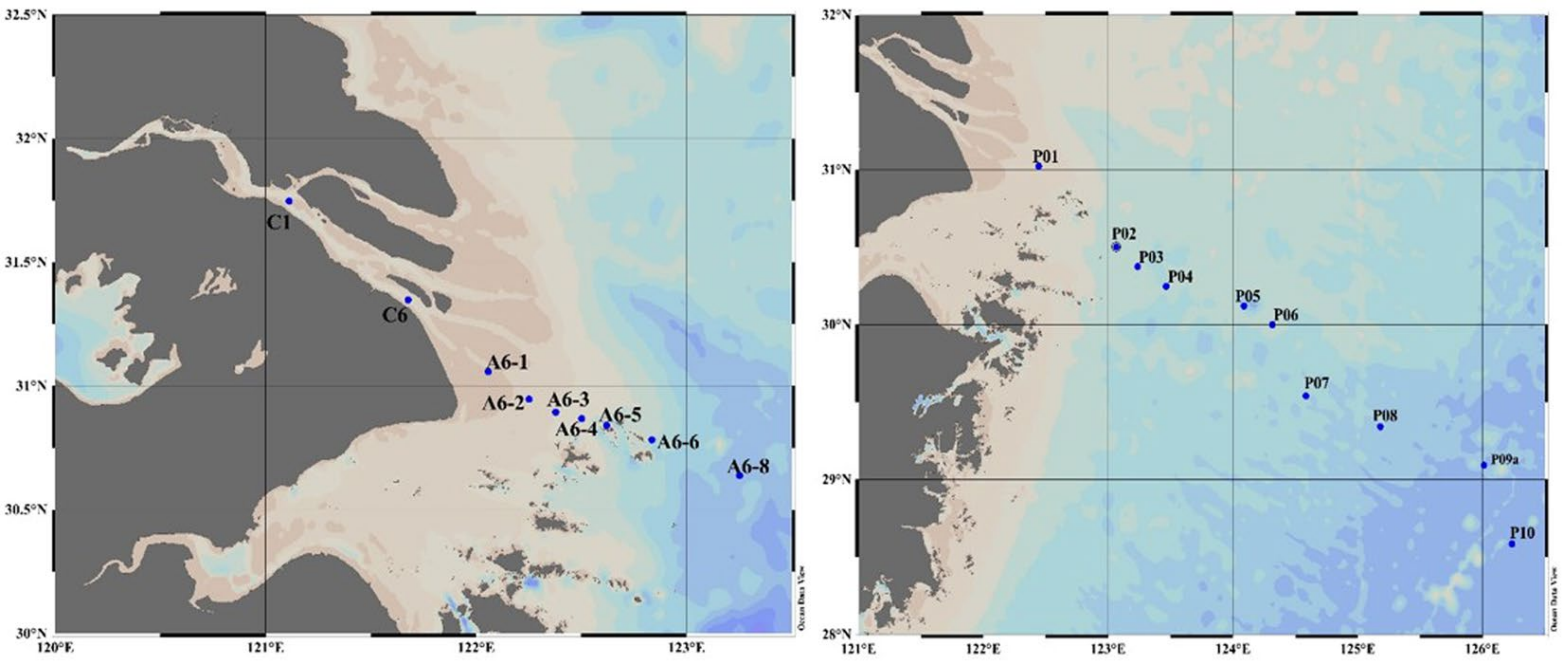
Locations of the seawater sampling stations in the Yangtze River estuary (July, 2015; left panel) and the ECS (October, 2011; right panel). (Ocean Data View (ODV) software, version 4.5.6, Schlitzer, R., http://odv.awi.de, 2013).

In this study, samples from the YRE (Fig. 1) were collected using Niskin bottles internally lined with Teflon during a summer cruise of the R.V. “Run Jiang” (9–20 July, 2015). To compare the influence of the ECS on HS concentrations, we collected and analyzed HA and FA in high salinity samples from the ECS (October, 2011, P01–P10, Fig. 1).

Sampling, filtration, equipment, reagents, optimization of conditions for analysis of HA and FA concentrations in the YRE, and analysis of dFe concentrations are all described in the Supplementary Information.

## Results

### The dFe, FA and HA concentrations in the Yangtze River estuary

**Figure 2:**
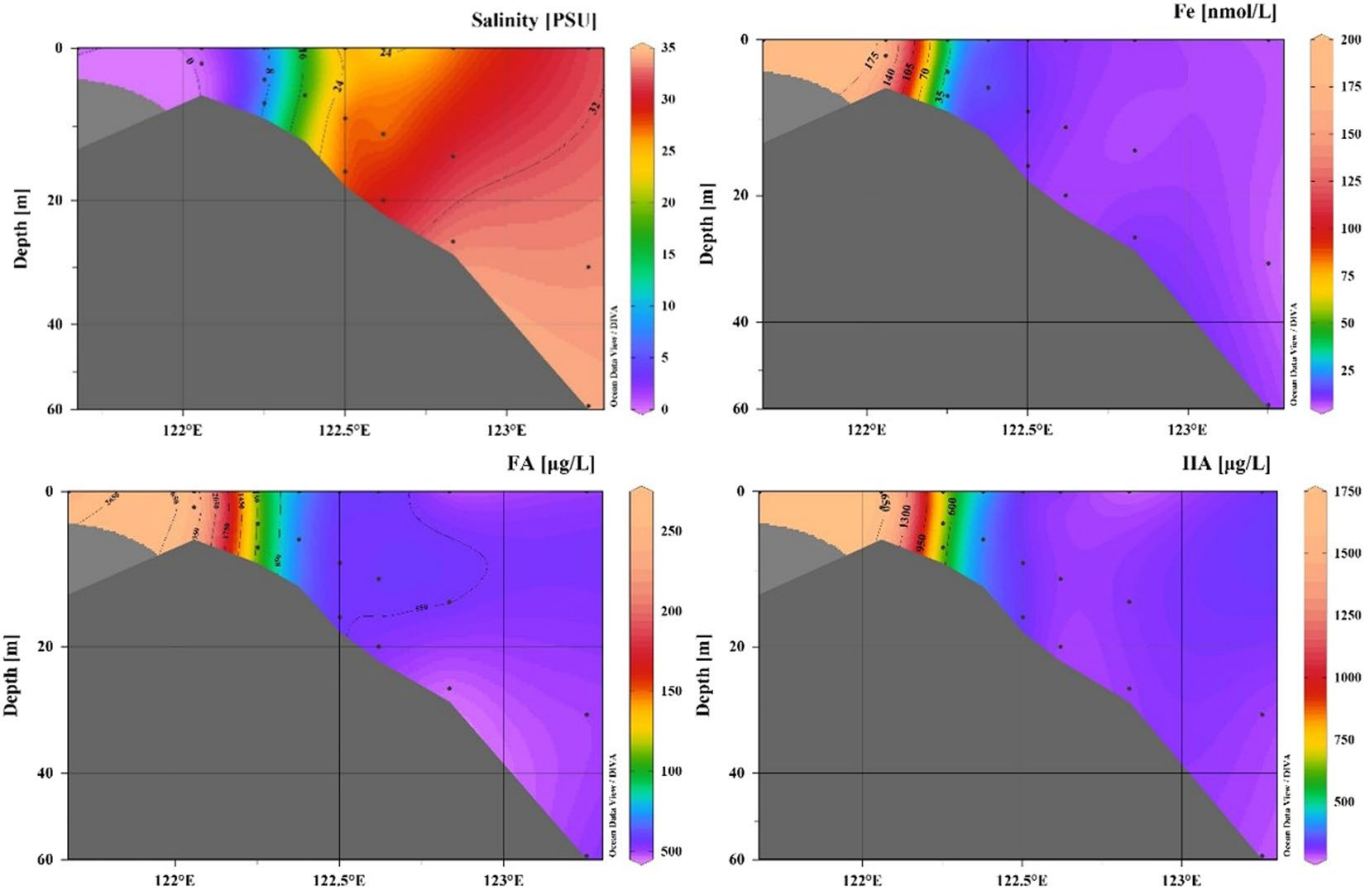
Vertical section of salinity, Fe concentration (nmol/L), FA (μg/L) and HA (μg/L) concentration along the Yangtze River estuary. (Ocean Data View (ODV) software, version 4.5.6, Schlitzer, R., http://odv.awi.de, 2013).

We determined the dFe, FA and HA concentrations of the YRE samples (Fig. 2). Along the salinity gradient (C6-A6–8, Fig. 1) from freshwater (salinity < 0.15) to the estuary (salinity > 34), dFe, FA and HA displayed similar decreasing trends (Fig. 2), and their values ranged from 176.5 to 6.3 nmol/L, 2510.4 to 461.6 μg/L and 1739.8 to 277.2 μg/L, respectively. The loss rates of dFe, FA and HA were up to 96%, 79%, and 84% (Table 1), respectively, which were consistent with the findings of previous studies^23, 26^. The dFe concentrations in Yangtze River freshwater at stations C1 and C6 were 175.9 nmol/L and 176.5 nmol/L, respectively (Table 1).

**Table 1:** dFe (nmol/L), FA (μg/L), HA (μg/L), and the IBC of FA and HA (cal, nmol Fe/(mg HS)) as determined from the YRE samples.

Station	Deep	Salinity	dFe (nmol/L)	FA (μg/L)	HA (μg/L)	IBC of FA	IBC of HA
C6	0	0.14	176.5	2423.2	1712.3	72.8	103.1
A6-1	0	0.68	163.5	2431.2	1682	67.3	97.2
	2	0.31	165.3	2510.4	1739.8	65.8	95
A6-2	0	7.28	37.44	1095.5	694.2	34.2	53.9
	4	6.57	30.7	1126.5	673.4	27.3	45.6
	7	7.76	25.8	1114.7	638.8	23.1	40.4
A6-3	0	16.38	19.57	750.1	448.4	26.1	43.6
	6	17.2	17.66	702.2	402.6	25.1	43.9
A6-4	0	25.29	10.91	573.1	320.6	19	34
	9	27.09	11.58	557.3	326	20.8	35.5
	16	27.34	10.04	558.8	328	18	30.6
A6-5	0	23.26	9.96	587.7	307	16.9	32.4
	11	26.9	9.18	569.8	301.9	16.1	30.4
	20	29.67	7.96	533.3	295.4	14.9	27
A6-6	0	26.86	8.28	488	277.2	17	29.9
	14	30.61	7.68	553	307.7	13.9	25
	26	33.02	10.18	461.6	300.9	22.1	33.8
A6-8	0	31.49	6.66	511.5	319.1	13	20.9
	30	33.46	6.28	495.6	296.8	12.7	21.2
	59	34.19	8.78	514.3	285.6	17.1	30.7
The total removal rate			96%	79%	84%		

### The dFe, FA and HA concentrations along section P in the ECS

**Figure 3:**
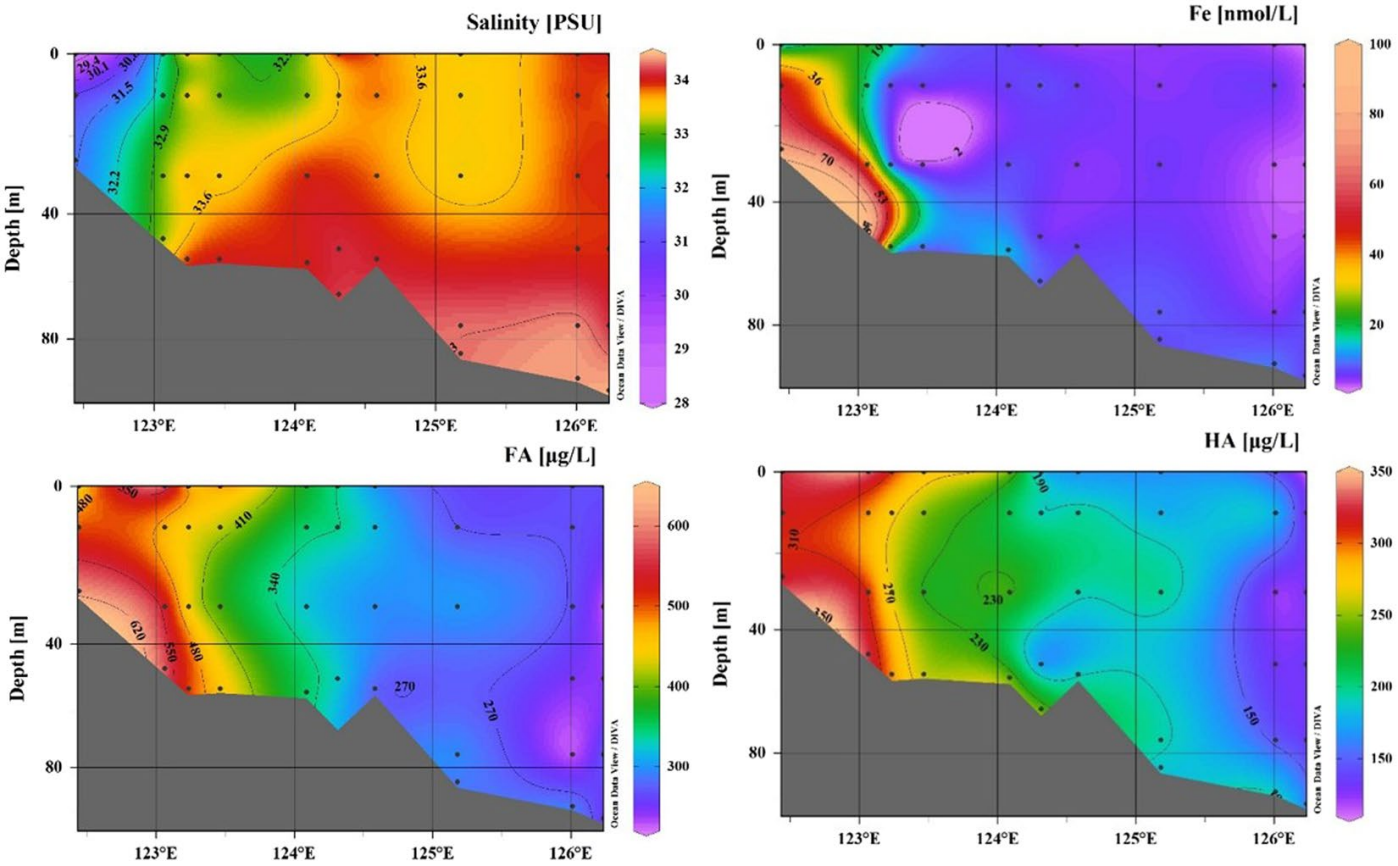
Vertical section of salinity and Fe (nmol/L), FA (μg/L) and HA (μg/L) concentrations along section P in the ECS. The Fe concentrations were obtained from Su *et al*. (2015)^44^, and the salinity data were obtained from Li *et al*. (2014)^50^. (ODV software, version 4.5.6, Schlitzer, R., http://odv.awi.de, 2013).

The dFe, FA and HA concentrations with decreasing salinities along the P section of the ECS are shown in Fig. 3. Along this salinity gradient (28.09–34.44, Fig. 3) from P01 to P10 (Fig. 3), dFe, FA and HA all exhibited similar trends, in which higher concentrations of dFe, FA, and HA were observed in the nearshore region than in the offshore region (Fig. 3), and they were in the ranges of 2.6 to 95.0 nmol/L, 223.8–608.9 μg/L, and 121.4–338.5 μg/L, respectively. The highest FA and HA concentrations were observed at the bottom of station P01 (608.9 μg/L) and the surface of station P02 (338.5 μg/L), whereas both of the lowest concentrations of FA and HA were observed at station P09a and were 223.8 μg/L and 121.4 μg/L, respectively.

### The numerical models for HS and Fe concentrations in YRE

Taking the baseline value of seawater into account, the relationship between the salinity gradient and the dFe, FA and HA concentrations could be described by a first-order exponential removal model, in which salinity is the only independent variable:

1 $${\rm{y}}({\rm{dFe}}/{\rm{FA}}/{\rm{HA}},{\rm{S}})={{\rm{a}}}_{0}\times \exp (kS)+{y}_{0}$$

where y (dFe/FA/HA, S) is the amount of dFe, FA or HA remaining at salinity S; a_0_ is the removal amount of dFe (nmol/L), FA (μg/L) or HA (μg/L) at the maximum salinity of the YRE; *k* is the attenuation coefficient of dFe, FA and HA concentrations with salinity; S is the salinity of the water ranging from 0 to 34; and y_0_ is the total amount of dFe (nmol/L), FA (μg/L) or HA (μg/L) at maximum salinity. Furthermore, the sum of a_0_ and y_0_ is theoretically the amount of dFe (nmol/L), FA (μg/L) or HA (μg/L) at salinity 0 in this model.

According to the data obtained at the Yangtze River estuary (Table 1) and from fitting the exponential equation (1), the attenuation function for dFe, FA and HA with increasing salinity could be described with the following equations:

2 $${\rm{y}}({\rm{dFe}})=175.28\times \exp (-0.28\times {\rm{S}})+9.86$$

3 $${\rm{y}}({\rm{FA}})=2044.5\times \exp (-0.16\times {\rm{S}})+522.0$$

4 $${\rm{y}}({\rm{HA}})=1503.0\times \exp (-0.19\times {\rm{S}})+305.2$$

**Figure 4:**
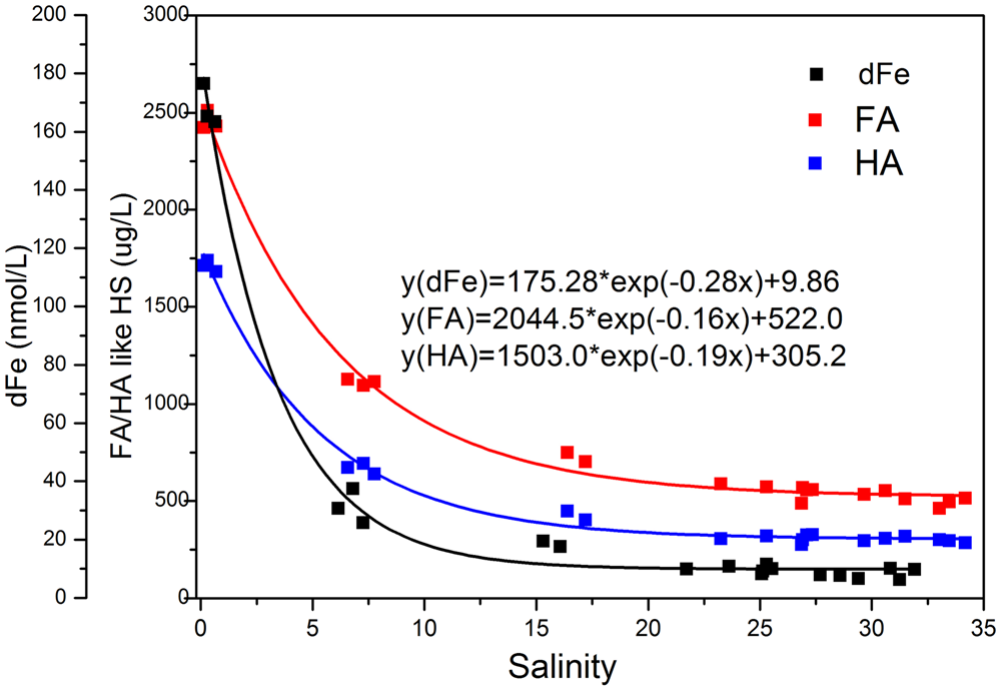
Non-linear curve fit to dFe, FA and HA concentrations along the salinity gradient, and the relationship to removal of dFe, FA and HA along the salinity gradient. “x” represents the salinity of the YRE.

The attenuation functions for dFe, FA and HA in YRE as described with equations 2, 3 and 4 were all well fitted with equation 1, and their correlation coefficients (R^2^) were 0.9939, 0.9936 and 0.9950, respectively (P < 0.05). The parameter “*k*” is different because factors such as the river flow velocity, dFe concentration and DOM (or HS) content at different estuaries and seas/oceans may be different. This first-order exponential removal model only considers the influence of salinity in the area where freshwater mixes with seawater and is only suitable for the estuary. These fitting equations confirm that dFe and HS co-varied in the estuary (Fig. 4).

According to the above exponential removal model equation 1, we can calculate the maximum removal rates of dFe, FA and HA in YRE with the following equation:

5 $$Y(the\,removal\,of\,dFe/FA/HA)=(1-\frac{{y}_{0}}{{a}_{0}+{y}_{0}})\times 100 \% $$

where Y is the removal percentage of the total dFe, FA or HA.

The removal rates of dFe, FA and HA obtained from equations 2, 3, 4 and 5 were 94.7%, 79.7% and 84%, respectively, which were similar to the removal rates obtained from the field data (Table 1).

### The IBC in UVSW and standard seawater at different salinities

**Figure 5:**
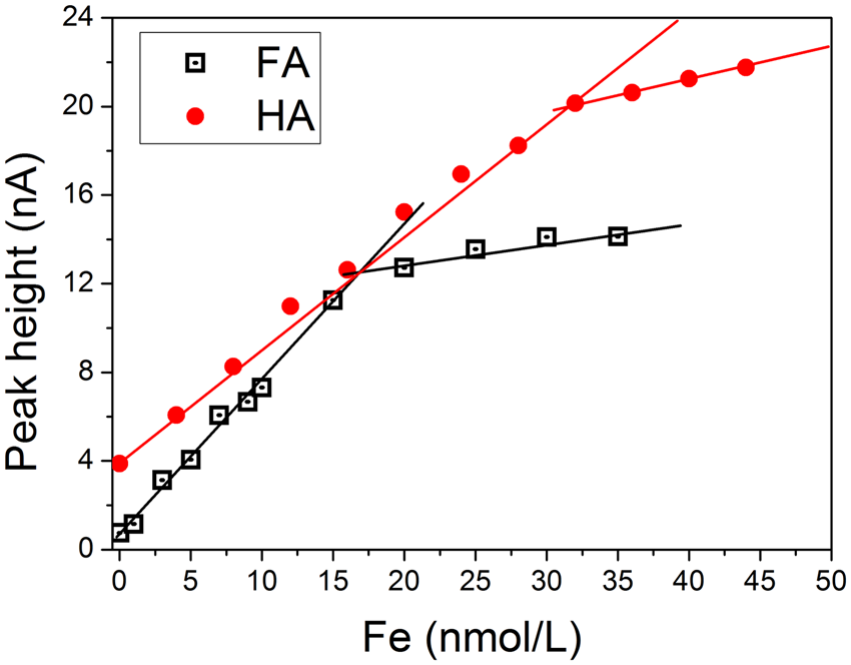
Iron binding capacity of FA and HA in the UVSW in the presence of 1 mg/L HS.

The IBC value of FA and HA (Fig. 5) could be obtained using the value of total dissolved iron (dFe, nmol) divided by the FA/HA concentration (mg) of the samples (equation i) (ref. 36):

6 $${\boldsymbol{IBC}}=\frac{{\boldsymbol{dFe}}({\boldsymbol{nmol}}\cdot {{\boldsymbol{L}}}^{-1})}{{\boldsymbol{HS}}({\boldsymbol{mg}}\cdot {{\boldsymbol{L}}}^{-1})}$$

The IBC in the UVSW was determined by titrating nmol Fe to 1 mg of FA and HA in UVSW. Figure 5 shows that IBC of FA and HA in the UVSW were 16.34 ± 0.40 nmol Fe/﻿(mg FA) and 32.07 ± 1.75 nmol Fe/﻿(mg HA) (n = 4), respectively (Table 2).

**Table 2:** IBC of SRHS as a function of salinity based on laboratory analysis. Every IBC value of SRHS at different salinities was measured more than four times, and its average (AVG) and standard deviation (SD) are displayed.

Salinity		5	12.5	20	30	35	40	UVSW
FA	AVG	25.2	23.4	20.6	16.9	16.8	16.8	16.3
	SD	1.4	1.9	2.1	0.4	1.6	1.3	0.4
HA	AVG	41.4	38.3	35.7	33.5	31.5	31.5	32.1
	SD	1.6	2.1	1.5	1.5	1.6	1.6	1.7

We also measured the IBC of SRHS in a series of standard seawater samples with different salinities; the results are shown in Table 2. The IBC of SRHS decreased with increasing salinity from 5 to 40, ranging from 25.21 to 16.80 nmol Fe/mg for FA and from 41.40 to 30.50 nmol Fe/mg for HA (Table 2). The IBC values obtained by this study in the salinity range of 30 to 40 were in good agreement with those of Laglera *et al*. (2007)^38^ and Laglera and van den Berg (2009)^11^, which were 16.7 ± 2.0 nmol Fe/﻿(mg FA) and 32 ± 2.2 nmol Fe/﻿(mg HA), respectively.

### The numerical models for the IBC of HA and FA at different salinities

From equations 1 and the definition of IBC (equation 6), the decrease of IBC along with the salinity gradient could be described by the following exponential equation:

7 $$Y(IBC,S)={A}_{o}\times exp(kS)+{Y}_{0}$$

where Y (IBC, S) is the IBC value of the HS (FA/HA) of natural samples at salinity S; K is the attenuation coefficient of the salinity and IBC; A_0_ is the total reduction amount of the IBC of FA (μg/L) or HA (μg/L) at the maximum salinity of the YRE; S is the salinity of the water ranging from 0 to 34; and Y_0_ is the minimum IBC value at maximum salinity. Furthermore, the sum of A_0_ and Y_0_ is theoretically the total IBC of FA (μg/L) or HA (μg/L) at salinity 0 in this model.

**Figure 6:**
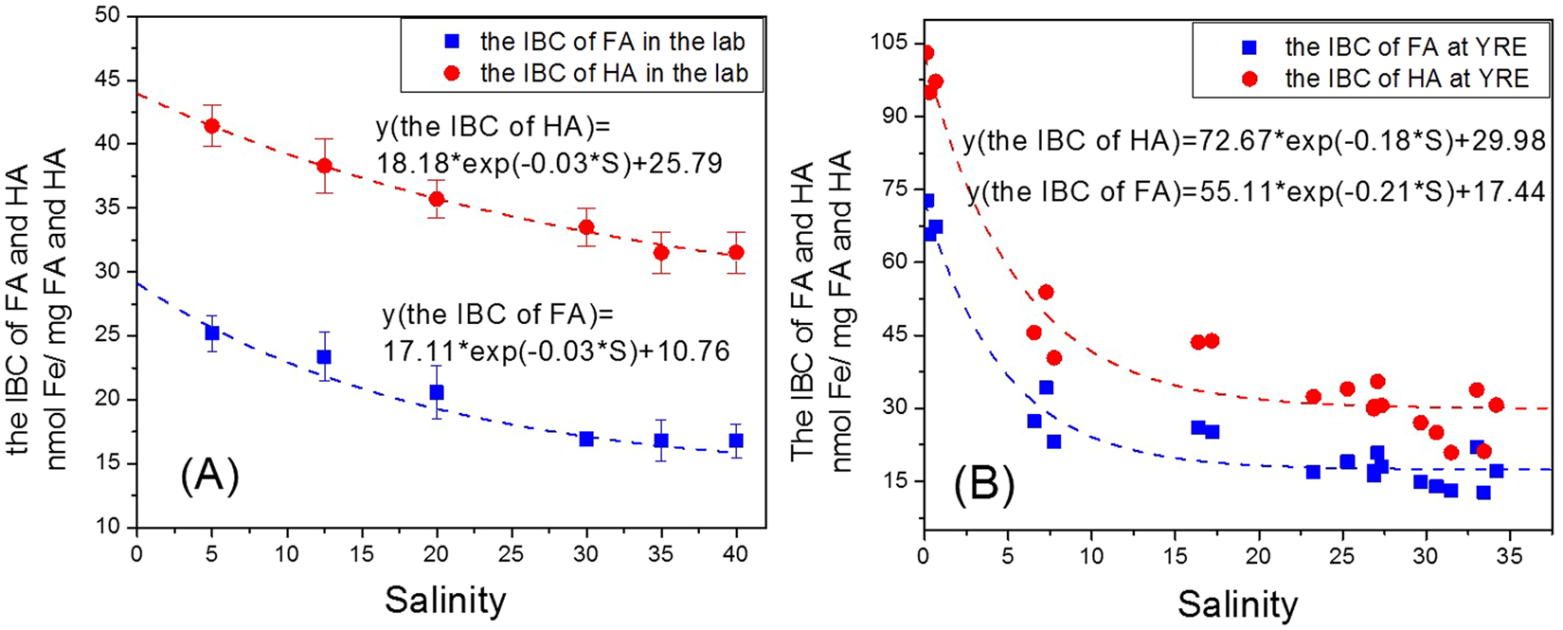
Effect of salinity on the iron binding capacity of FA/HA of the SRFA/HA (**A**) and Yangtze River estuarine samples (**B**). The dashed line represents the fitting curves fit to first-order exponential equations. (**A**) The error bars around the IBC of SRFA/HA at every salinity value of standard seawater were standard deviations of the average values of IBC.

In our study, the variation of IBC for SRFA and HA obtained from standard seawater series (salinity of 5, 12.5, 20, 30, 35 and 40) showed a non-linear decrease with salinity (Fig. 6A). The IBC of FA decreased from 25.21 to 16.93 nmol Fe/(﻿mg FA) along with the salinity gradient from 5 to 40 which could be described by the following equation 8 (R^2^ = 0.948, P < 0.05, Fig. 6A):

8 $$Y(the\,IBC\,of\,FA,S)=17.11\times exp(\,-\,0.03\times S)+10.76$$

The same situation was observed for SRHA-iron complexation in this salinity. The IBC of HA decreased from 41.40 to 31.50 nmol Fe/﻿(mg HA) along the salinity gradient of 5 to 40, which could be described by equation 9. Then it remained stable from a salinity of 35 to 40 (R^2^ = 0.987, P < 0.05, Fig. 6A):

9 $$Y(the\,IBC\,of\,HA,S)=18.18\times exp(\,-\,0.03\times S)+25.7$$

### The IBC at the Yangtze River Estuary

The IBC results along the salinity gradient of the Yangtze River estuary indicated that the IBC of natural HS decreased with increasing salinity (Table 1). The highest IBC was observed at station C6, where salinity was only 0.14; the IBC values for FA and HA were 72.8 nmol Fe/mg and 103.1 nmol Fe/mg respectively. At stations A6–8, the surface salinity was 31.49, and the IBC values decreased to 13.0 nmol Fe/﻿(mg FA) and 20.9 nmol Fe/﻿(mg HA), respectively, which were lower than those obtained in the laboratory (Table 1).

Our IBC results along the salinity gradient at YRE indicated that the IBC of natural HS at YRE could be modeled by equation 7, which is shown in Fig. 6B (R^2^ (the IBC of FA) = 0.954 and R^2^ (the IBC of HA) = 0.942, both P < 0.05; equation 9 and 10, respectively).

10 $${\rm{y}}({\rm{the}}\,{\rm{IBC}}\,{\rm{of}}\,{\rm{FA}},{\rm{S}})=55.11\times \exp (-0.21\times {\rm{S}})+17.44$$

11 $${\rm{y}}({\rm{the}}\,{\rm{IBC}}\,{\rm{of}}\,{\rm{HA}},{\rm{S}})=72.67\times \exp (-0.18\times {\rm{S}})+29.88$$

According to equations 10 and 11, the maximum IBC of FA and HA at salinity 0 were 72.55 nmol Fe/﻿(mg FA) and 102.55 nmol Fe/﻿(mg HA), which were similar to the data obtained at stations C6 and C1 of the YRE (Table 1).

## Discussion

### The removal of dFe, FA and HA in the YRE and ECS

The concentrations of dFe, FA and HA decreased with salinity in both the YRE and ECS. The total decrease rates for dFe, HA and FA at YRE were 96%, 84% and 79%, respectively, while in ECS they were 95.0%, 63.2% and 64.1%, respectively. The removal of dFe, FA and HA mainly occurred in the mixing area, where the salinity increased from 0.14 to 17 (Fig. 4); after the mixing area, where salinity increased from 17 to 34, removal slowed down. The behavior of dFe, FA and HA along the salinities 17 to 34 was consistent with the findings of Mahmood *et al*. (2015) for the Mersey River estuary^22^. HS and dFe are known to co-precipitate across the salinity range of 16.38 to 34.18 in estuarine systems^22^ (Fig. 4).

The removal of HA and FA was due to flocculation in the estuary where fresh water mixed with seawater^20, 29^. There, the ionic strength increased quickly, and as most of the humic substances (HS) were colloids^30–32^, a huge quantities of ions with positive charge resulted in the flocculation of HS^28^.

This removal rate of dFe in estuaries is consistent with previous studies of Boyle *et al*. (1977)^29^ and Dai and Martin (1995)^40^, according to whom loss rates of dFe were 96% (Millica River estuary) and 98% (Yenisty River estuary). The flocculation of HS in the estuaries was responsible for removal of dFe^40–42^. As for the situation at section P in the ECS, the actual removal rate of dFe, FA and HA are higher than the estimates. So, there may be another reason such as uptake by phytoplankton^25^, or water current movement, that accounts for the observations. In the high primary productivity of the ECS, dFe would be taken up by phytoplankton, especially in summer and during algal blooms^43, 44^. Furthermore, movement of currents in the ECS would influence the variation of dFe and HS from near-shore to offshore. For example, the Taiwan Warm Current coming from the Kuroshio would carry low dFe and HS waters into the ECS and decrease the dFe and HS concentrations in the ECS^44, 45^.

The removal rate of dFe was much higher than those of HA and FA (Table 1). The removal rate of iron in both this study and previous research^29, 40, 46, 47^ were all above 95%. Thus, there should be other reasons that account for the removal of more dFe than HS in estuaries. Our results showed that a decrease of the IBC with increasing salinity may be responsible for part of that (Fig. 4). The IBC of FA and HA along the salinity gradient showed a first order decrease along with increased salinity (Table 1).

Recent work has shown that some special functional groups, such as O,O-, O,N- and O,S-coordination motifs in the HS molecules, could complex with iron and other metal ions^48^. The degree of Fe-HA complexation is greatly influenced by the change of the HA structure in different ionic strengths (0–0.234 g/L NaCl)^37^. Increasing ionic strength lowers the electrostatic repletion among its functional groups and enables HA to fold and form a more compact shape^37, 49^. Furthermore, it has been suggested that the complexation ability of HA in freshwater is much higher than at a salinity of 5 in standard seawater. The possible reason might be the difference between SRHA/FA and DOM in YRE. Another reason could be that experimental salinities of standard seawater start form 5 only, while most removal already took place in the salinity range 0–5. At the natural pH of freshwater, the complexation ability of HA may be higher than 133.3 nmol Fe/mg HA due to the more deprotonated HA^37^. In our study, the IBC of FA/HA in freshwater (station C6) reached a maximum level (Table 1). In the estuaries, mixture of freshwater with seawater increases the ionic strength of water; therefore, the structure of HS was more compact along the increasing salinity gradient, which might be responsible for the smaller IBC value at higher salinities (Fig. 6).

Furthermore, the calcium and magnesium concentrations in seawater were much higher than those in freshwater. Although the complexation of calcium and magnesium with HS were much weaker than with HS-Fe, the high concentrations of calcium and magnesium in seawater competed with iron for HS. This may be one of reasons that the IBC of SRFA/HA decreased along the salinity gradient.

### The numerial model of dFe, FA and HA in other global estuaries

The first-order exponential attenuation model (equation 1) can describe the decrease in dFe, FA and HA with increasing salinities well. The removal rates of dFe, HA and FA calculated by this model were similar to those from previous research in other estuary areas^29, 40, 46, 47^. In order to test whether our model could be used in other areas, we applied our exponential model to other estuaries, such as the Ob River estuary^40^, Yenisey River estuary^40^, Galveston Bay^47^, Millica River estuary^29^, Zaire River estuary^46^ and San Francisco Bay estuary^50^, which were studied previously. We use data from these previous studies of dFe and HS along a salinity gradient and fit these data with our exponential model (Table 3). The results showed that our exponential model could also be used for other areas; the R^2^ values were all higher than 0.98. With this model, we can get the concentrations of dFe, FA and HA at every salinity value with only a few data points, and this was especially valuable at places where it is difficult to sample. These results have confirmed that our exponential model could be applied in other estuaries (Table 3).

**Table 3:** Exponential curve equations of the dFe and HS concentrations using data obtained from a previous study and our results.

Species	Study Area	Fitted Equation	R-square (R^2^)	Salinity range	Removal rate	Number of points	Data Sources
dFe	Ob River estuary	y = 552.73*exp(−0.16 S) + 8.50	0.9948	1.3–34	99%	7	ref. 40
dFe	Yenisey River estuary	y = 240.99*exp(−0.15 S) + 3.64	0.9996	0–34	99%	7	ref. 40
dFe	Galveston Bay	y = 418.23*exp(−0.31 S) + 5.40	0.9985	0–33.9	97%	9	ref. 47
dFe	Millica River estuary	y = 24379.0*exp(0.32 S) + 757.2	0.9951	0–29	99%	16	ref. 29
dFe	Zaire River estuary	y = 232.84*exp(−0.14 S) + 16.61	0.9825	0–31	97%	8	ref. 46
HS	San Francisco Bay	y = 7190.3*exp(−0.66 S) + 41.33	0.9892	4.0–34	94%	5	ref. 51
dFe	Yangtze River estuary	y = 175.28*exp(−0.28 S) + 9.86	0.9939	0–34	96%	20	This study
FA	Yangtze River estuary	y = 2044.5*exp(−0.16 S) + 422.0	0.9936	0–34	79%	20	This study
HA	Yangtze River estuary	y=1503.0*exp(−0.19 S)+305.2	0.9950	0–34	84%	20	This study

“S” represents the salinity of the estuary and “y” represents is the dFe/HS/FA/HA content remaining at salinity S.

The parameters of k, a_0_ and y_0_ may be different in different areas, because dFe and HS at different estuaries have their own biogeochemical cycles. For example, in San Francisco Bay, water enters the California continental shelf, which is close to the open Pacific ocean; and the concentrations of dFe and HS decrease quickly, while the salinities increase quickly, so the value of attenuation efficient, “k” (−0.66 for HS) in this area was higher than that of YRE (−0.19 for HA) (Table 3). This is because Yangtze River water flows into the ECS and is mostly constrained to the continental self, leading to high dFe concentrations^14, 25^. Thus, the parameters “k” and “a_0_” are much higher in San Francisco Bay than in the YRE, whereas “y_0_” is much lower (Table 3). The equations in Table 3 suggested that the removal of dFe and HS along the salinity gradient in different estuaries could be expressed using the same exponential curves (equation 1).

In conclusion, we note three points. First, FA and HA decreased along the salinity gradient following an exponential removal trend in the estuary. Second, the IBC of FA/HA along the salinity gradient decreased with an exponential removal pattern marked by a slow removal rate, k (the IBC of FA/HA). Third, the minimum concentrations (y_0_ in the model) of dFe, HA and FA could be obtained by the model we derived in this study.

## Electronic supplementary material

S.I. of Capacity of humic substances to complex with iron at different salinities in the Yangtze River estuary and East China Sea
