# Supplementary material for: Capacity of humic substances to complex with iron at different salinities in the Yangtze River estuary and East China Sea
Source: Sci Rep. 2017 May 3;7:1381. doi: 10.1038/s41598-017-01533-6 (PMC5431113; doi:10.1038/s41598-017-01533-6)
Supplement: Supplementary file 1 — S.I. of Capacity of humic substances to complex with iron at different salinities in the Yangtze River estuary and East China Sea [file 41598_2017_1533_MOESM1_ESM.doc]

**Capacity of humic substances to complex with iron at different salinities in the Yangtze River estuary and East China Sea**

**Rujun Yang*, Han Su, Shenglu Qu, Xuchen Wang**

**Supplementary Figures:**

Supplementary Fig. 1. Effect of the drop size on the peak height in the presence of 50 nmol/L Fe and 3.0 mg/L FA or 3.0 mg/L HA.

|  |  |
| --- | --- |

Supplementary Fig. 2. Effect of pH on the peak height in the presence of 20 mmol/L bromate, 50 nmol/L Fe and 2.0 mg/L HA; (A) pH from 8.81 to 7.03; (B) pH from 6.57 to 8.96.

Supplementary Fig. 3. Effect of deposition potential on the peak height of 1.5 mg/L FA/HA in ECSW in the presence of 20 nmol/L bromate and 40 nmol/L Fe.

Supplementary Fig. 4. Equilibrium time for the formation of Fe-FA/HA at pH 8.15.

|  |  |
| --- | --- |

Supplementary Fig. 5. (A) Linear range of HS in which HS could be determined in the presence of 20 nmol/L Fe; (B) Linear range of HS in which HS could be determined in the presence of 40 nmol/Fe.

**Methods**

**Measurement**

**Sampling.** Temperature and salinity were measured in situ using conductivity, temperature and depth (CTD) sensors. After collection, the samples were pressure-filtered through acid-cleaned 47-mm filter-holders loaded with 0.4-μm Nuclepore membranes (Millipore Company, Ireland). The membranes were treated as per Su et al. (2015)^1^. The filtered seawater was collected in cleaned LDPE sample bottles; one bottle was acidified to pH <2 using HCl (Suprapur, Merck, Germany) for the analysis of dFe; the others were frozen for subsequent analysis of FA and HA. The dFe concentrations in the YRE samples were measured according to the methods described in Su et al. (2015)^1^.

**Equipment.** Total dissolved iron, HA and FA were analyzed by the CSV method. The voltammetric system used a 797 VA computrace (Metrohm, Switzerland) instrument with a Teflon automatic agitator, a hanging mercury drop electrode (HMDE, Metrohm model 797 VA, Switzerland) as the working electrode, Pt as the auxiliary electrode and a double junction (Ag/AgCl, 3 mol/L KCl) as the reference electrodes. The instrument was controlled using 797 VA computrace software. Samples (10 mL) were placed in a PTFE voltammetric cell, purged with high-purity nitrogen gas and stirred for 300 s. A reducing current of Fe-HS complexes was generated, and each reduction current was measured 3 times. All of the procedures and the 797 VA computrace were performed in a class-100 clean laminar flow bench (ESCO, Singapore). UVSW was used to optimize the CSV response of the Fe-FA/HA species. Seawater from the East China Sea (filtered through 0.2-μm Nuclepore membranes (Millipore Company, Ireland), a low dFe concentration) was UV-digested for 12 h using a home-built system with a 400 W, high-pressure, mercury vapor lamp in a 300-mL quartz cup, referred to as UVSW (salinity 34).

**Reagents.** Sample and reagent bottles (LDPE, Nalgene) were immersed in a tank containing 0.1% detergent for 3 days in Milli-Q water, rinsed with Milli-Q water 6 times, immersed in a tank containing the 10% HCl mass fraction (Guaranteed reagent, Sinopharm, China) for 3 days, rinsed with Milli-Q water 6 times, immersed in another tank of 10% HCl (Guaranteed reagent, Sinopharm, China) overnight, rinsed with Milli-Q water, and then filled with MQ water that was acidified using HCl (Suprapur, Merck) to pH 2. The reagents, including BrO_3_^−^ (0.4 mol/L)/POPSO (0.1 mol/L), 1μmol/L and 10 μmol/L Fe(NO_3_)_3_, 0.1 mol/L DHN (2,3-dihydroxynaphthalene), and 3 mol/L KCl, were all made in water (Milli-Q water, 18.2 MΩ) according to Su et al. (2015)^25^. FA and HA stock solutions (1 g/L) were prepared in MQ water from standard reagents of SRFA and SRHA (Suwannee River, International Humic Substances Society, IHSS, SRFA 1S101F and SRHA 2S101H, called FA and HA). All of the stock solutions were kept in the dark at 4 ℃ for no longer than one month. Man-made standard seawater samples (China Series Standard Seawater, National Center of Ocean Standards and Metrology, Tianjin) with different salinities and low trace metal and HS contents were used to determine the IBC of SRFA/HA at different salinities, including 5.007, 12.504, 19.998, 29.999, 35.002, and 39.999, respectively.

**The optimized conditions for determining FA and HA**

FA and HA were determined by CSV-monitored iron titrations according to the method of Laglera et al. (2007)^2^. We changed the CSV parameters to obtain the optimal conditions, as our samples were collected from ECS, which contained high iron and organic ligand concentrations. Both FA and HA, which represented HS-like substances, were determined by two types of standard substances: SRFA and SRHA.

**Optimization of the mercury drop size.** The instrument parameter-drop size controlled the mercury drop size, which affected the sensitivity of the CSV response. Additions of Fe and FA/HA into 10 mL of UVSW (final concentration, 50 nmol/L Fe and 3.0 mg/L FA/HA) in the presence of 20 mmol/L bromate showed that the voltammetric response linearly increased with drop size (Supplementary Fig. 1). However, increasing the drop size decreased the stability of the mercury drop. A drop size of 4 was found to be a good compromise between the sensitivity of the CSV response and the stability of the mercury drop.

**Optimization of the pH.** The pH was varied by adding small amounts of HCl to UVSW containing 50 nmol/L Fe and 2.0 mg/L HA, adjusting the pH from 8.81 to 7.03, and the CSV response was simultaneously measured for Fe-HA species, showing strong agreement with Laglera et al. (2007)^2^ (Supplementary Fig. 2 A). Furthermore, adding a small amount of NH3•H2O to the UVSW containing the same concentration of iron and HS would change the pH of the system. The CSV response for a Fe-HA species was tested by changing the pH from 6.57 to 8.96 (Supplementary Fig. 2 B). It could be observed that the reductant current was strongly pH dependent due to the deprotonation of HA and the hydrolysis of Fe ^3^. The variation of these two curves may be due to the instability of Fe-HS species or the poor solubility in high pH water hydrolysis of iron during the experiment^1^. In general, a higher voltammetric response was observed at high pH (pH>8.1). Therefore, it is convenient to maintain the pH in the range of 8.1 to 8.2 to achieve high sensitivity and an appropriate linear range.

**Effect of varying the deposition potential.** To measure the CSV response for Fe-HA/FA species at different deposition potentials, seawater samples from the ECS (ECSW) containing 40 nmol/L Fe and 1.5 mg/L HA/FA were measured immediately (Supplementary Fig. 3). The highest sensitivity of the Fe-HA and Fe-FA response was found in the range from -0.05 to -0.25 V. Finally, the highest Fe-HA and Fe-FA responses were observed at deposition potentials of -0.2 and -0.05 V, respectively, which were chosen for our analysis of deposition potential.

**Optimal equilibration time.** We added 20 nmol/L Fe and 1.0 mg/L HA/FA at pH of 8.15 to ECSW or UVSW together; the peak heights of the Fe-HA/FA species were measured after a certain time (Supplementary Fig. 4). The reduction current was found to stabilize after a reaction time of 20 min; in other words, natural HS in seawater was fully complexed by iron (Supplementary Fig. 4). Therefore, in our study, we chose 0.6-1.5 h as our equilibration time. We first added 50 nmol/L Fe to ECSW, after 1 h of equilibration. Then 2.0 mg/L FA/HA was added into the same seawater sample, and the CSV response for Fe-HS was measured immediately, which represented the stability of the free (uncomplexed) HS complexing with iron after a certain time.

We optimized the conditions for the Chinese coastal sea, including the differential pulse mode (pulse amplitude of 0.05 mV for FA and 0.03 mV for HA (Supplementary Fig. 3), with a pulse time of 0.02 s, achieving the best linear range of FA/HA), with a drop size of 4 (Supplementary Fig. 1), deposition potential of -0.05 V for FA and -0.2 V for HA (Supplementary Fig. 4), pH of 8.1-8.2 (Supplementary Fig. 2), deposition time of 60 s, scan rate of 90 mV/s, and equilibration time of 0.6-1.5 h (Supplementary Fig. 4) in the presence of 40 nmol/L Fe and 20 mmol/L bromate. The detection limits were 14.1 μg/L HA (n=20) and 16.4 μg/L FA (n=14). The concentration of FA and HA in samples were determined by the optimized CSV method. Therefore, HS could be accurately represented by both FA and HA, which could be determined to complement each other. The FA and HA of the low salinity samples of the YRE were measured and diluted to the same salinity of the UVSW.

**The linear range of FA and HA in the UVSW.** The maximal combination of FA and HA additions to the UVSW in the presence of 20 nmol/L Fe increased linearly up to 1.2 and 0.9 mg/L, respectively (Supplementary Fig. 5 A). Though these values might be sufficient for open seawater, they may be insufficient for some coastal waters and estuarine waters, such as the YRE and ECS, as the total iron concentration may be up to 40 nmol/L (ref. 1). Therefore, we added 40 nmol/L Fe in the UVSW to enhance the response of the FA/HA concentrations. These maximal combinations of FA and HA in the presence of 40 nmol/L iron increased linearly up to 2.5 and 1.8 mg/L, respectively (Supplementary Fig. 5 B).

**Reference:**

1. Su, H., Yang, R., Zhang, A. ＆ Li. Y. Dissolved iron distribution and organic complexation in the coastal waters of the East China Sea. Mar. Chem. **173,** 208-221 (2015).
2. Laglera, L. M., Battaglia, G. ＆ van den Berg, C. M. G. Determination of humic substances in natural waters by cathodic stripping voltammetry of their complexes with iron. Anal. Chim. Acta **599(1),** 58-66 (2007).
3. Fang, K. et al. Effect of environmental factors on the complexation of iron and humic acid. Journ. Environ. Sci. 27, 188-196 (2015).
